# Supplementary material for: Resolving the full spectrum of human genome variation using Linked-Reads
Source: Genome Res. 2019 Apr;29(4):635–45. doi: 10.1101/gr.234443.118 (PMC6442396; doi:10.1101/gr.234443.118)
Supplement: Supplemental Material [file supp_gr.234443.118_Supplemental_longranger-2.2.zip › longranger-2.2.tar.bz2!/lr2.2-public/lib/stan/cmdstan/stan/doc/api/html/structstan_1_1math_1_1coupled__ode__system_3_01_f_00_01double_00_01stan_1_1agrad_1_1var_01_4.html]

Stan: stan::math::coupled\_ode\_system< F, double, stan::agrad::var > Struct Template Reference


|  |  |
| --- | --- |
|  | Stan  2.5.0  probability, sampling & optimization |


- Main Page
- Related Pages
- Namespaces
- Classes
- Files

- Class List
- Class Index
- Class Hierarchy
- Class Members

All Classes Namespaces Files Functions Variables Typedefs Enumerations Enumerator Friends Macros Pages

- stan
- math
- coupled\_ode\_system< F, double, stan::agrad::var >

Public Member Functions |
Public Attributes |
List of all members

stan::math::coupled\_ode\_system< F, double, stan::agrad::var > Struct Template Reference

The coupled ODE system for known initial values and unknown parameters.
More...

`#include <coupled_ode_system.hpp>`

|  |  |
| --- | --- |
| Public Member Functions | |
|  | coupled\_ode\_system (const F &f, const std::vector< double > &y0, const std::vector< stan::agrad::var > &theta, const std::vector< double > &x, const std::vector< int > &x\_int, std::ostream \*msgs) |
|  | Construct a coupled ODE system with the specified base ODE system, base initial state, parameters, data, and a message stream. More... |
|  | |
| void | operator() (const std::vector< double > &y, std::vector< double > &dy\_dt, double t) |
|  | Assign the derivative vector with the system derivatives at the specified state and time. More... |
|  | |
| int | size () const |
|  | Returns the size of the coupled system. More... |
|  | |
| std::vector< double > | initial\_state () |
|  | Returns the initial state of the coupled system. More... |
|  | |
| std::vector< std::vector  < stan::agrad::var > > | decouple\_states (const std::vector< std::vector< double > > &y) |
|  | Returns the base ODE system state corresponding to the specified coupled system state. More... |
|  | |

|  |  |
| --- | --- |
| Public Attributes | |
| const F & | f\_ |
|  | |
| const std::vector< double > & | y0\_dbl\_ |
|  | |
| const std::vector  < stan::agrad::var > & | theta\_ |
|  | |
| std::vector< double > | theta\_dbl\_ |
|  | |
| const std::vector< double > & | x\_ |
|  | |
| const std::vector< int > & | x\_int\_ |
|  | |
| const int | N\_ |
|  | |
| const int | M\_ |
|  | |
| const int | size\_ |
|  | |
| std::ostream \* | msgs\_ |
|  | |

## Detailed Description

### template<typename F> struct stan::math::coupled\_ode\_system< F, double, stan::agrad::var >

The coupled ODE system for known initial values and unknown parameters.

If the base ODE state is size N and there are M parameters, the coupled system has N + N \* M states.

The first N states correspond to the base system's N states:

The next M states correspond to the sensitivities of the parameters with respect to the first base system equation:

The final M states correspond to the sensitivities with respect to the second base system equation, etc.

Template Parameters
:   |  |  |
    | --- | --- |
    | F | type of functor for the base ode system. |

Definition at line 63 of file coupled\_ode\_system.hpp.

## Constructor & Destructor Documentation

template<typename F >

|  |  |  |  |
| --- | --- | --- | --- |
| stan::math::coupled\_ode\_system< F, double, stan::agrad::var >::coupled\_ode\_system | ( | const F & | *f*, |
|  |  | const std::vector< double > & | *y0*, |
|  |  | const std::vector< stan::agrad::var > & | *theta*, |
|  |  | const std::vector< double > & | *x*, |
|  |  | const std::vector< int > & | *x\_int*, |
|  |  | std::ostream \* | *msgs* |
|  | ) |  |  |

inline

Construct a coupled ODE system with the specified base ODE system, base initial state, parameters, data, and a message stream.

Parameters
:   |  |  |  |
    | --- | --- | --- |
    | [in] | f | the base ODE system functor. |
    | [in] | y0 | the initial state of the base ode. |
    | [in] | theta | parameters of the base ode. |
    | [in] | x | real data. |
    | [in] | x\_int | integer data. |
    | [in,out] | msgs | stream to which messages are printed. |

Definition at line 88 of file coupled\_ode\_system.hpp.

## Member Function Documentation

template<typename F >

|  |  |  |  |  |  |
| --- | --- | --- | --- | --- | --- |
| std::vector<std::vector<stan::agrad::var> > stan::math::coupled\_ode\_system< F, double, stan::agrad::var >::decouple\_states | ( | const std::vector< std::vector< double > > & | *y* | ) |  |

inline

Returns the base ODE system state corresponding to the specified coupled system state.

Parameters
:   |  |  |
    | --- | --- |
    | y | coupled states after solving the ode |

Definition at line 219 of file coupled\_ode\_system.hpp.

template<typename F >

|  |  |  |  |  |
| --- | --- | --- | --- | --- |
| std::vector<double> stan::math::coupled\_ode\_system< F, double, stan::agrad::var >::initial\_state | ( |  | ) |  |

inline

Returns the initial state of the coupled system.

Because the initial values are known, the initial state of the coupled system is the same as the initial state of the base ODE system.

This initial state returned is of size `size()` where the first N (base ODE system size) parameters are the initial conditions of the base ode system and the rest of the initial condition elements are 0.

Returns
:   the initial condition of the coupled system.

Definition at line 204 of file coupled\_ode\_system.hpp.

template<typename F >

|  |  |  |  |
| --- | --- | --- | --- |
| void stan::math::coupled\_ode\_system< F, double, stan::agrad::var >::operator() | ( | const std::vector< double > & | *y*, |
|  |  | std::vector< double > & | *dy\_dt*, |
|  |  | double | *t* |
|  | ) |  |  |

inline

Assign the derivative vector with the system derivatives at the specified state and time.

The input state must be of size `size()`, and the output produced will be of the same size.

Parameters
:   |  |  |  |
    | --- | --- | --- |
    | [in] | y | state of the coupled ode system. |
    | [out] | dy\_dt | populated with the derivatives of the coupled system at the specified state and time. |
    | [in] | t | time. |

Exceptions
:   |  |  |
    | --- | --- |
    | exception | if the system function does not return the same number of derivatives as the state vector size. |

Definition at line 123 of file coupled\_ode\_system.hpp.

template<typename F >

|  |  |  |  |  |
| --- | --- | --- | --- | --- |
| int stan::math::coupled\_ode\_system< F, double, stan::agrad::var >::size | ( |  | ) | const |

inline

Returns the size of the coupled system.

Returns
:   size of the coupled system.

Definition at line 187 of file coupled\_ode\_system.hpp.

## Member Data Documentation

template<typename F >

const F& stan::math::coupled\_ode\_system< F, double, stan::agrad::var >::f\_

Definition at line 65 of file coupled\_ode\_system.hpp.

template<typename F >

const int stan::math::coupled\_ode\_system< F, double, stan::agrad::var >::M\_

Definition at line 72 of file coupled\_ode\_system.hpp.

template<typename F >

std::ostream\* stan::math::coupled\_ode\_system< F, double, stan::agrad::var >::msgs\_

Definition at line 74 of file coupled\_ode\_system.hpp.

template<typename F >

const int stan::math::coupled\_ode\_system< F, double, stan::agrad::var >::N\_

Definition at line 71 of file coupled\_ode\_system.hpp.

template<typename F >

const int stan::math::coupled\_ode\_system< F, double, stan::agrad::var >::size\_

Definition at line 73 of file coupled\_ode\_system.hpp.

template<typename F >

const std::vector<stan::agrad::var>& stan::math::coupled\_ode\_system< F, double, stan::agrad::var >::theta\_

Definition at line 67 of file coupled\_ode\_system.hpp.

template<typename F >

std::vector<double> stan::math::coupled\_ode\_system< F, double, stan::agrad::var >::theta\_dbl\_

Definition at line 68 of file coupled\_ode\_system.hpp.

template<typename F >

const std::vector<double>& stan::math::coupled\_ode\_system< F, double, stan::agrad::var >::x\_

Definition at line 69 of file coupled\_ode\_system.hpp.

template<typename F >

const std::vector<int>& stan::math::coupled\_ode\_system< F, double, stan::agrad::var >::x\_int\_

Definition at line 70 of file coupled\_ode\_system.hpp.

template<typename F >

const std::vector<double>& stan::math::coupled\_ode\_system< F, double, stan::agrad::var >::y0\_dbl\_

Definition at line 66 of file coupled\_ode\_system.hpp.

---

The documentation for this struct was generated from the following file:

- src/stan/agrad/rev/ode/coupled\_ode\_system.hpp


---

[ Stan Home Page ]

*© 2011–2014,
Stan Development Team.*
